# Inconsistent advice by ChatGPT influences decision making in various areas

**DOI:** 10.1038/s41598-024-66821-4

**Published:** 2024-07-10

**Authors:** Shinnosuke Ikeda

**Affiliations:** Human and Social Administration Department, Kanazawa University, Kanazawa University Kakuma-machi, Kanazawa, Ishikawa 920-1192 Japan

**Keywords:** ChatGPT, Decision making, Moral judgment, Delay discounting, Gender stereotype, Personal fear of invalidity, Psychology, Human behaviour

## Abstract

The ChatGPT technology is increasingly becoming a part of our daily lives and is starting to be utilized in various decision-making contexts. The current study builds upon prior research, demonstrating that people’s moral decision-making is influenced by ChatGPT across three perspectives, as evidenced by two studies (total n = 1925). The findings suggested that ChatGPT advice impacted decision-making similarly to expert advice, although not all decisions were susceptible to influence, particularly those based on negative emotions. Additionally, ChatGPT advice affected decisions beyond moral judgments, but no effect was observed when the advice recommended immediate low rewards. Moreover, individuals with a higher tendency for personal fear of invalidity were more likely to be influenced by both expert and AI advice, but this was not related to trust in AI.

## Introduction

Every day, we engage in a variety of decision-making processes that span different aspects of our lives, from choosing what to eat at restaurants^1^ to selecting schools for education^2^ to planning home renovations^3^. While some decisions are made independently, others are influenced by advice from others^4^. Research has shown that when making decisions based on others' advice, higher quality advice is given more weight^5^, and conversely, individuals tend to discount the importance of the advice when they are confident in their own decision-making abilities^6^. Neuroscience studies also indicate that the advisor's expertise and the discrepancy between one's own opinion and the content of the advice are integrated when making decisions based on advice^7^.

Advice in decision-making can be provided by human experts or by ChatGPT, which has rapidly emerged in recent years^8^. ChatGPT is a chatbot powered by advanced machine learning software developed by OpenAI, trained with various texts and images sourced from the Internet, and responds with appropriate words and phrases in response to user queries^9^. ChatGPT has integrated into our lives so swiftly that its advice is often sought in many aspects of our daily activities^10^. Although ChatGPT advice can be potentially erroneous^11^, it is sometimes rated superior to human expert advice^12^.

As ChatGPT increasingly becomes a part of our lives, understanding the extent to which our decision-making can be influenced by ChatGPT's advice is crucial. Krügel et al.^13^ highlighted that people's moral judgments are influenced by the advice of ChatGPT. In their study, the focus was on the Trolley Problem, which questions the morality of sacrificing one person to save five others. Opinions stating that the answer to the Trolley Problem is either right or wrong were generated by ChatGPT, and participants were presented with these opinions and asked to respond to the Trolley Problem. Additionally, the conditions were set to present the advice as either generated by ChatGPT or by a human moral advisor. The results showed that the number of responses indicating that the action was right increased when the "right" opinion was presented, and the number of responses indicating that the action was wrong increased when the "wrong" opinion was presented, regardless of whether the advice was presented as coming from a human advisor or ChatGPT. In other words, humans are influenced by the advice of ChatGPT even in moral decision-making.

The present study expands on the findings of Krügel et al.^13^ in three significant ways. First, Krügel et al.^13^ did not include a control condition without any advice, leaving it unclear whether the right or wrong advice impacted decision-making in both scenarios. Therefore, this study incorporates a control condition where no advice is given and examines the influence of advice provided either by ChatGPT or by an expert. It is important to note that while this study involves Japanese participants, research indicates that there are no cultural differences in ethical decision-making regarding the trolley problem between Asia and the West^14–16^.

Second, this study aims to determine whether ChatGPT advice similarly influences decision-making in contexts other than moral decision-making. It remains uncertain whether the influence of ChatGPT advice on decision-making is confined to the moral domain or extends to other areas as well. Consequently, decision-making in various domains will be further explored in an exploratory study. Additional decision-making tasks in this study focus on delayed value discounting and gender stereotypes. Delayed value discounting refers to the phenomenon where the subjective value of rewards to be received in the future is perceived as less than their immediate value^17^. For instance, if someone chooses "$8000 obtained immediately" over "$10,000 obtained in five years," it indicates that the value of the monetary reward is perceived as discounted by more than $2000 due to the five-year delay^18^. This study will investigate the influence of ChatGPT advice on economic decision-making, specifically in the context of delayed value discounting. Regarding gender stereotypes, the focus will be on the medical field. It has been observed that female doctors, particularly in the surgical field where women are less represented, are more likely to receive lower and harsher ratings than male doctors^19,20^. The present study will examine the impact of ChatGPT advice by creating a task that challenges stereotypes about surgeons, thereby assessing whether ChatGPT advice can influence perceptions related to gender stereotypes in the medical profession.

Third, this study will explore the personal characteristics that determine susceptibility to advice from ChatGPT. Specifically, the study will focus on Personal Fear of Invalidity as a decision-making style and Trust in Artificial Intelligence. Personal Fear of Invalidity refers to the tendency to experience difficulty in making decisions in daily life and to reconsider decisions made later. Indeed, it has been demonstrated that individuals with high Personal Fear of Invalidity are more prone to regret decisions they have made and to consider a broad spectrum of possibilities when making decisions^21^. In this study, it was hypothesized that individuals with higher levels of Personal Fear of Invalidity would be more susceptible to influence from AI and expert advice. Trust in Artificial Intelligence measures the trust in the social benefit and fidelity of AI. It has been demonstrated that individuals who exhibit higher Trust for Artificial Intelligence tend to have greater distrust in human selfishness^22^. In this study, it was hypothesized that the greater an individual’s Trust for Artificial Intelligence, the more they would be influenced by the advice provided by ChatGPT.

Building on the methodology of Krügel et al.^13^, this study seeks to expand on their findings in the three areas outlined above. Initially, Study 1 will explore the control condition without advice, and in this context, attempts will be made to condition a newly created delayed value discounting task and a gender stereotype task. Study 1 serves as an exploratory investigation aimed at establishing the problem statement for Study 2. Study 2 will present advice generated by ChatGPT and examine its effects on responses, as well as the influence of individual characteristics. If individuals are influenced by advice from ChatGPT across various domains, it is anticipated that participants' responses will also be swayed by such advice, with the possible exception of moral tasks. Moreover, the tendency to be influenced by this advice is likely to be shaped by individual characteristics, including decision-making style and trust in AI. The procedures for this study were approved by the Ethical Review Committee of the Institute of Human and Social Sciences, Kanazawa University (approval number 2023-65). All procedures performed in studies involving human participants were in accordance with the ethical standards of the author's affiliation and with the 1964 Helsinki Declaration and its later amendments or comparable ethical standards.

## Study 1

### Methods

#### Participants

This study was commissioned and conducted by GMO Research (https://gmo-research.jp/). Participants were Japanese individuals registered with GMO Research as monitors. The survey items were posted on the Internet, and participants responded to them by accessing a distributed URL. In total, 111 men and women aged 23–60 years (63 men and 48 women, mean age 46.8 years, standard deviation 9.30) responded to the survey. The sample size was communicated to GMO Research to be approximately 100 participants, similar to Krügel et al.^13^, and the data for the received responses were sent to the corresponding author. Participants were informed that participation in the study was voluntary and that the results would be used for research purposes and could be published after anonymization.

### Materials

In Study 1, participants engaged in four tasks. Initially, the Trolley Problem (switch) and the Trolley Problem (bridge) were presented. For these tasks, theme sentences were developed based on a previous study with Japanese subjects^23^. In the Trolley Problem (switch), participants were presented with a scenario where a runaway train with broken brakes is approaching five workers on the railway track. By pulling a lever at hand, the runaway train can be switched to a secondary track, saving the five workers but sacrificing one worker on the secondary track. Participants were then asked to choose whether they thought it was right to pull the lever and sacrifice one worker to save the five workers. In the Trolley Problem (bridge), the situation is similar up to the point where the runaway train is approaching the five workers, but this time there is no secondary track, and the respondents are on a bridge spanning the tracks. A person is standing next to the respondent, who can save the five workers by pushing the person off the bridge. However, in this case, the person who is pushed off the bridge will be sacrificed. Participants were then asked to answer in a binary choice whether they thought the act of pushing one person off was right or not. In the subsequent delayed value discounting task, participants were faced with 19 questions requiring them to choose between an immediate reward and a reward after one year. They responded by selecting one of the two options. The structure of the choices was as follows: initially, nine different scenarios were presented where the reward after one year was fixed at 100,000 yen, while the immediate reward varied from 10,000 yen to 90,000 yen in increments of 10,000 yen. Subsequently, ten additional scenarios were posed where the immediate reward was fixed at 100,000 yen, and the reward after one year ranged from 10,000 yen to 100,000 yen, also in 10,000 yen increments. The sequence in which these 19 questions were presented was randomized.

In the final gender stereotyping task, participants were presented with a scenario in which a malignant tumor is found during a physical examination, necessitating urgent surgical intervention. They were then asked to choose their preferred surgeon from 7 sentences comparing "a male surgeon with 30, 25, 20…5, 1 years of experience" and "a female surgeon with 30 years of experience," and from 6 sentences comparing "a male surgeon with 30 years of experience" and "a female surgeon with 25, 20…5, 1 year of experience." The sequence in which these 13 questions were presented was randomized.

#### Procedure

URLs for survey participation were distributed to monitors by GMO Research from January 15 to 17, 2024. Participants accessed the URL on their respective devices and responded to four tasks. In the delayed value discounting task and the gender stereotyping task, the order in which the conditional statements were presented was randomized.

#### Coding

In the two trolley dilemmas, participants were evaluated based on their moral judgment regarding the decision to save five people at the cost of one. Participants received 1 point for agreeing with and choosing the action to save five people at the sacrifice of one ("right" choice), and 0 points if they disagreed, deeming it wrong ("wrong" choice). In the delayed value discounting task, participants earned 1 point for opting for the immediate reward ("immediate" choice) and 0 points for selecting the delayed reward after one year ("after one year" choice). In the gender stereotyping task, 1 point was awarded for choosing a male surgeon ("male" choice) and 0 points for choosing a female surgeon ("female" choice).

### Results

**Figure 1:**
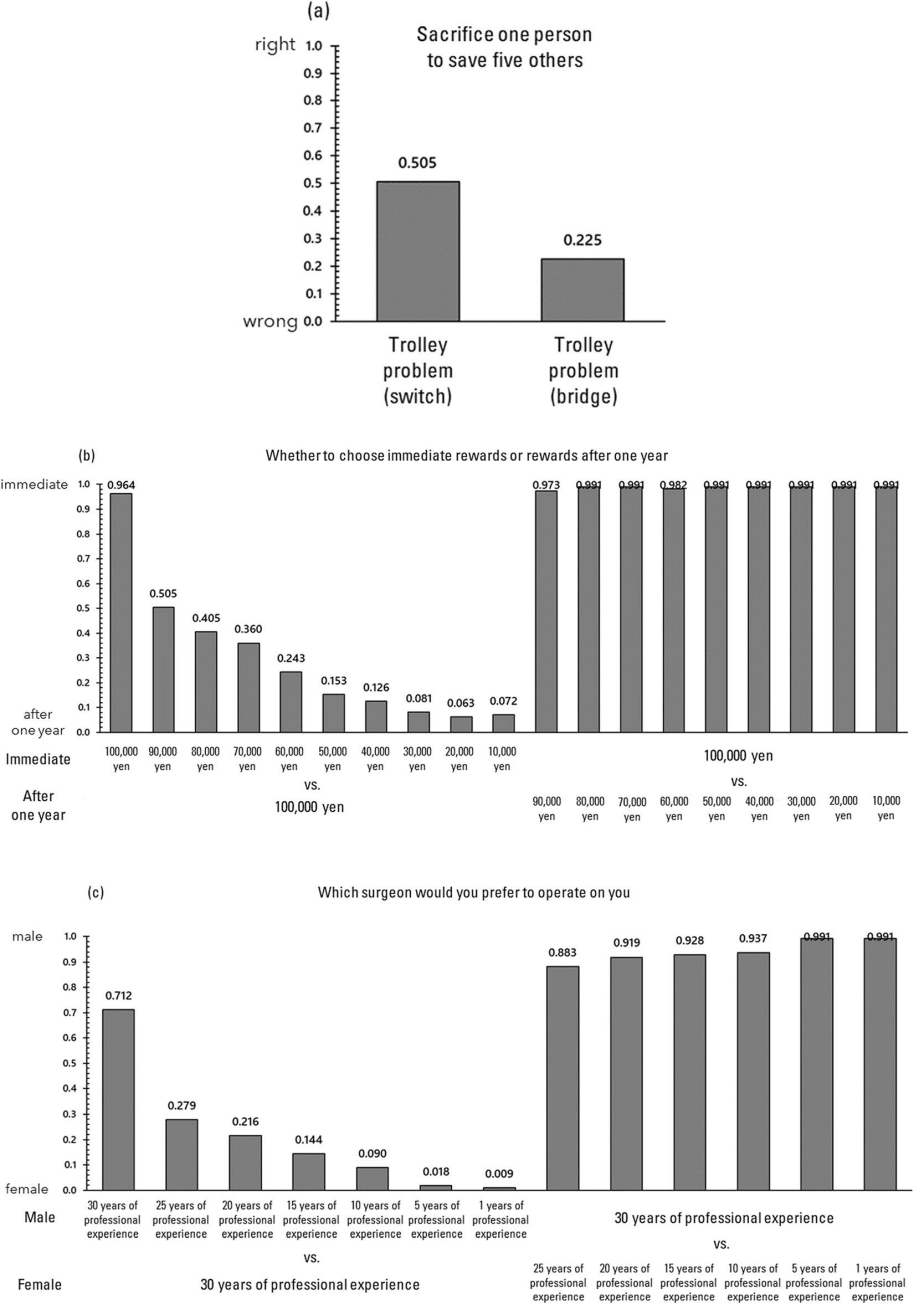
The response ratios for each task in Study 1.

The response ratios for the four tasks are illustrated in Fig. 1. Examining the response ratios for the trolley problem (Fig. 1a), a larger percentage of respondents in the switch condition (50.5%) compared to the bridge condition (22.5%) concurred that sacrificing one individual to save five was a correct decision. In the delayed value discounting task (Fig. 1b), the majority of respondents favored the immediate reward when the amounts of the immediate and 1-year-delayed rewards were identical, yet the proportion of respondents opting for the delayed reward escalated as the immediate reward diminished. Lastly, in the gender stereotype task (Fig. 1c), the majority of participants selected the male surgeon (71.2%) when both male and female surgeons had equivalent years of experience, but the proportion of participants choosing the female surgeon increased as the male surgeon's experience decreased.

### Discussion

Study 1 aimed to assess the control condition devoid of advice and to establish conditions for the newly devised delayed value discounting and gender stereotyping tasks in preparation for Study 2, which will investigate the impact of advice from ChatGPT. In the trolley problem, respondents were more inclined to sacrifice one individual to save five in the switch condition than in the bridge condition. This pattern aligns with a prior study^15^ and is believed to be influenced by the fact that the act of pushing off from the bridge elicits stronger negative emotions compared to switching the lever^24^. In the delayed value discounting task, the value of the delayed reward was perceived as discounted, consistent with previous studies^25^. This phenomenon is thought to be due to the reflection of individual impulsivity^26^. In the final gender stereotype task, male surgeons were more likely to be preferred when male and female surgeons had the same amount of years of experience, which may reflect the strictness with which female surgeons are evaluated^20^.

It is now essential to establish the conditions for the delayed value discounting task and the gender stereotype task for Study 2. This study will utilize the same proportions of responses to the trolley problem and alignment, specifically "90,000 yen immediately and 100,000 yen one year later (50.5%: the analogous 50.5% in the trolley problem (switch))" for the delayed value discounting, and "male surgeon with 20 years of experience and female surgeon with 30 years of experience (21.6%: the similar 22.6% in the trolley problem (bridge))" for the gender stereotype task were selected.

## Study 2

### Methods

#### Participants

Study 2, analogous to Study 1, was commissioned to GMO Research (https://gmo-research.jp/), and the URL for survey participation was disseminated to monitors. Participants were Japanese individuals registered with GMO Research as monitors and who had not participated in Study 1. The survey items were made available on the Internet, and participants responded to them by accessing the provided URLs. Ultimately, responses were collected from 1814 individuals (1051 men and 763 women, mean age 46.8 years, standard deviation 9.67) within the age range of 20 to 60. In Study 2, there were 16 conditions, as delineated below, and participants were randomly assigned to one of them. The sample size per condition was communicated to GMO Research to be approximately 100 participants, akin to that of Krügel et al.^13^, and the data from the received responses were forwarded to the authors. Participants were informed that participation in the study was voluntary and that the results would be utilized for research purposes and could be published after anonymization.

### Materials

In addition to the same trolley problem (switch) and trolley problem (bridge) as in Study 1, a delayed value discounting task comparing "¥90,000 to be given immediately and ¥100,000 to be given one year later" and a gender stereotype task comparing "a male surgeon with 20 years of experience and a female surgeon with 30 years of experience" were employed.

In response to these queries, ChatGPT (GPT-4) was requested to generate comments endorsing or recommending each of the two options. A total of 24 responses were produced, three for each of the two answers to the four tasks, culminating in eight responses. Eight university students were enlisted to assess the persuasiveness of their opinions on a scale from 1 to 10, and the responses from ChatGPT were chosen such that the two answers to the four tasks, totaling eight responses, had similar rating values (M = 8.2). All comments and question texts employed were uploaded to the Open Science Framework (OSF). As a comment on the endorsement of sacrificing one person in a trolley dilemma, the following advice was provided: "From an ethical perspective, it is occasionally deemed acceptable to sacrifice a few to save more lives. This viewpoint stems from the philosophical concept of 'utilitarianism.' Utilitarianism strives to achieve the greatest happiness for the greatest number of people, and in this scenario, saving five lives at the expense of one is justified".

Additionally, the Japanese Personal Fear of Invalidity Scale (J-PFIS)^21^ was employed to assess participants' personal fear of invalidity, and the Trust Scale for Artificial Intelligence (TAI)^22^ was utilized to gauge trust in artificial intelligence. The J-PFIS is a Japanese adaptation of the PFIS^23^, comprising nine items rated on a seven-point scale, including statements like "I tend to have difficulty in making most decisions." The J-PFIS embodies a single factor, which has been demonstrated to correlate with an individual's propensity for regret^21^. The TAI encompassed two factors: Trust in the social benefit of AI and Distrust in the fidelity of AI, with responses provided on a seven-point scale for 10 items such as "AI is beneficial to society" and "AI sometimes lies." Trust in AI has been shown to be inversely correlated with trust in humans^22^.

#### Procedure

URLs for survey participation were distributed to monitors by GMO Research from February 6 to 9, 2024. Participants accessed the URL on their respective devices and responded to four tasks. For each of the four tasks, either ChatGPT or an expert provided comments recommending one of the two options (with the advice being identical for both ChatGPT and the expert). Consequently, there were 16 conditions (4 (type of task) × 2 (type of advisor) × 2 (direction of advice)), and participants were randomly assigned to one of them. In each task, a task statement was presented first, followed by an advice statement, and participants were asked to choose their own response. Participants were then asked to indicate their choice had the advice not been given. For the advisor, in the ChatGPT condition, participants were presented with the following introductory text: "Before choosing your answer, please read the following answer by ChatGPT, which is an AI chatbot that can respond like a human." In the expert condition, the introduction read: "Before selecting your answer, please read the following answer by an expert in ________." In the "____" section, "morality" was entered for the trolley task, "economics" for the delayed value discount task, and "medicine" for the gender stereotyping task. After completing these tasks, participants were asked to respond to two scales, the J-PFIS and the TAI.

#### Coding

For the four decision-making tasks, participants were assigned a score of 0 or 1 according to the same criteria as in Study 1. For the J-PFIS, the mean scores for the 9 items were calculated in accordance with a previous study ^21^, and this average was used as the J-PFIS score. Similarly, for the TAI, the mean scores of the items corresponding to each of the two factors were calculated as per a previous study^22^, resulting in a score for trust in social benefit and a score for distrust in fidelity.

### Results

**Figure 2:**
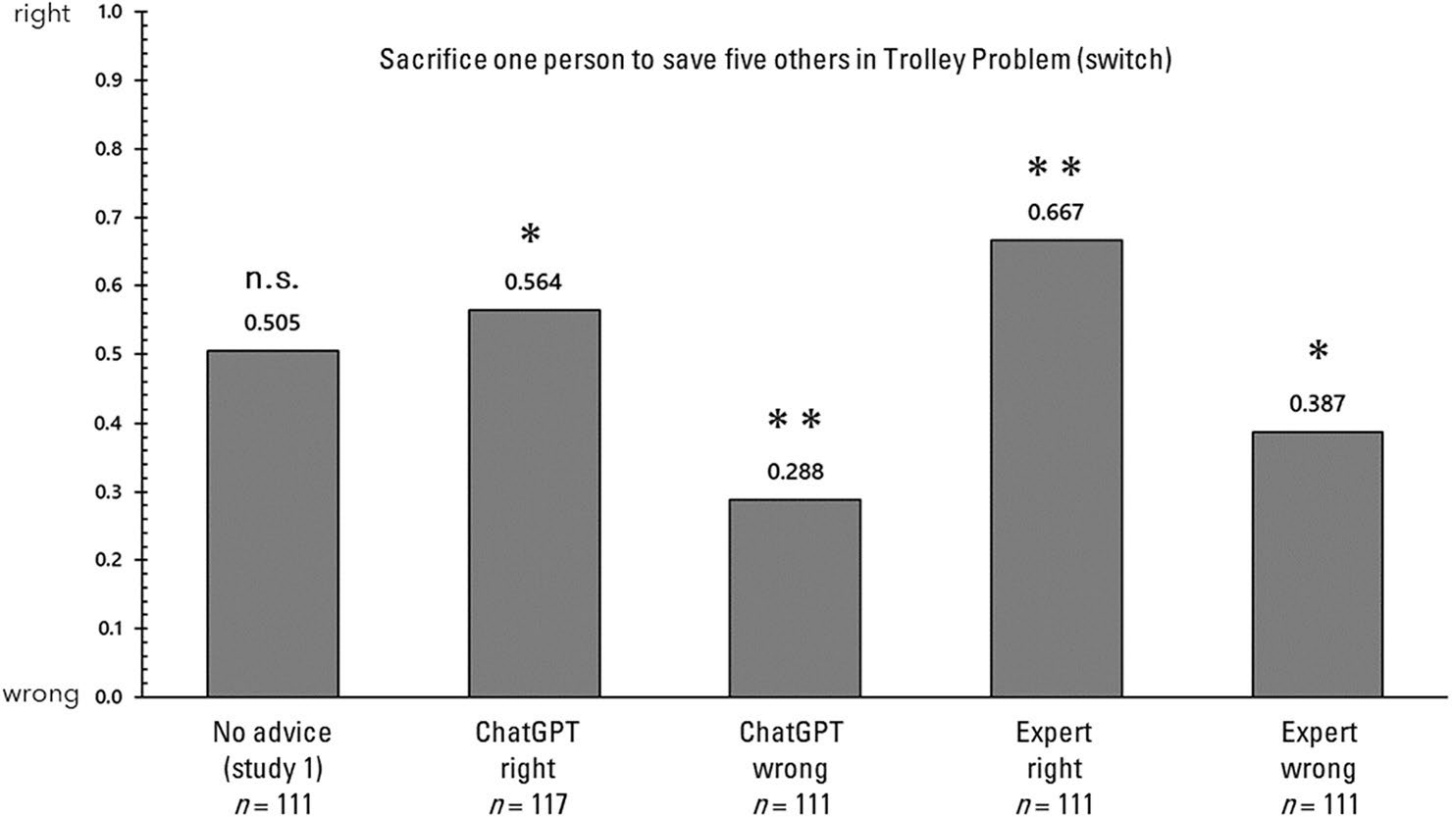
Response ratios for the trolley problem (switch) in Study 2. *p < 0.05, **p < 0.01.

**Figure 3:**
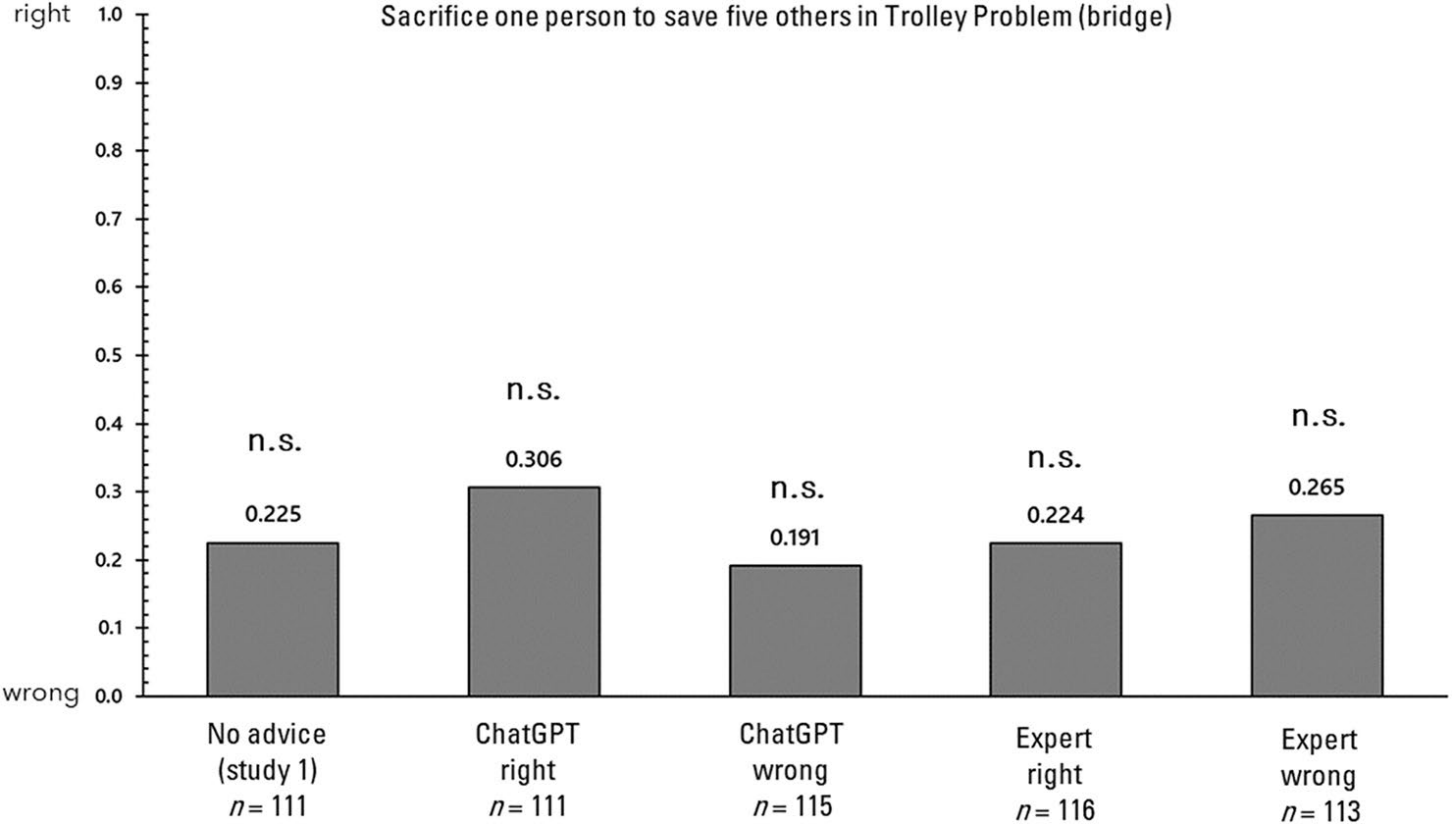
Response ratios for the trolley problem (bridge) in Study 2. *p < 0.05, **p < 0.01.

**Figure 4:**
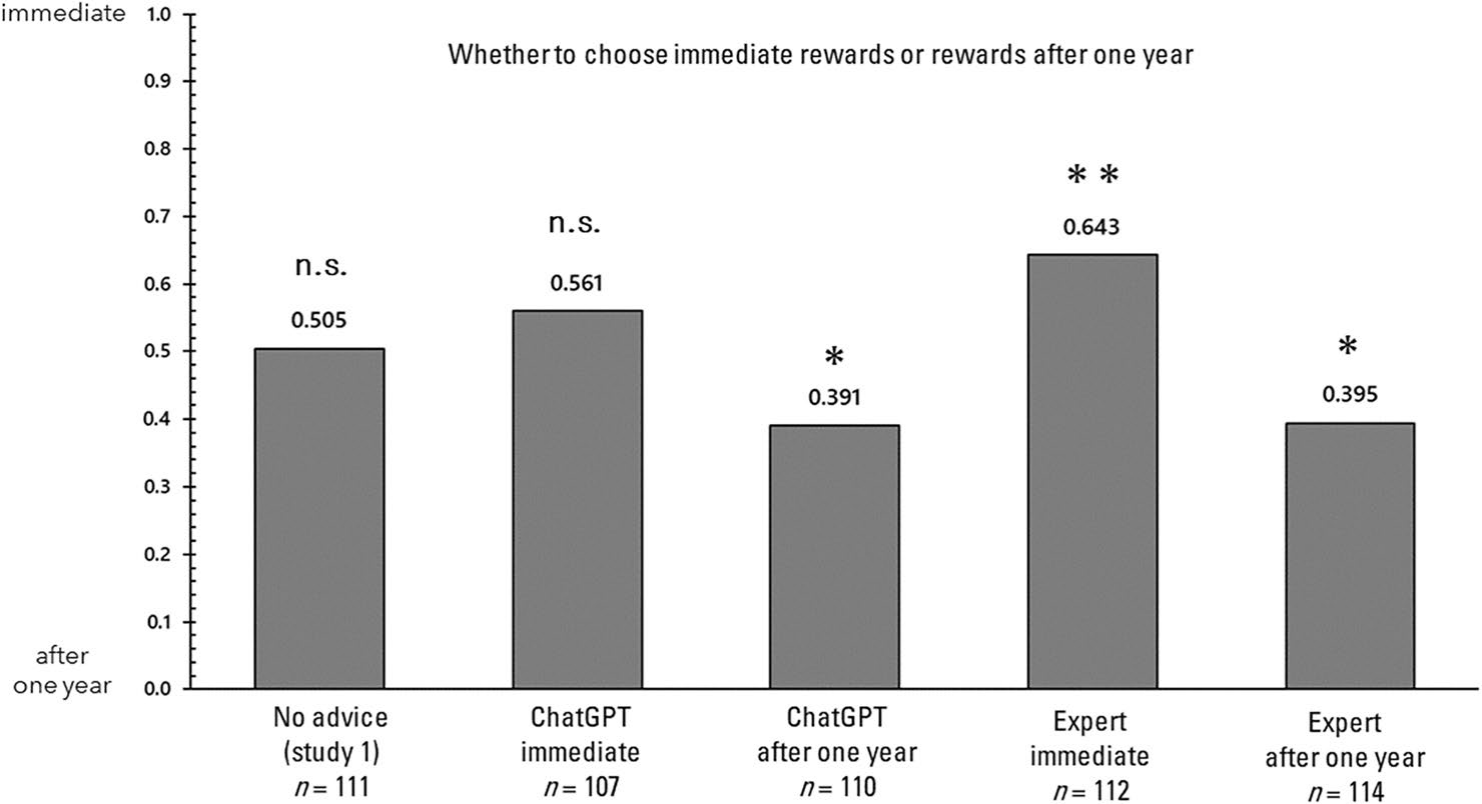
Response ratios for the delayed value discounting in Study 2. *p < 0.05, **p < 0.01.

**Figure 5:**
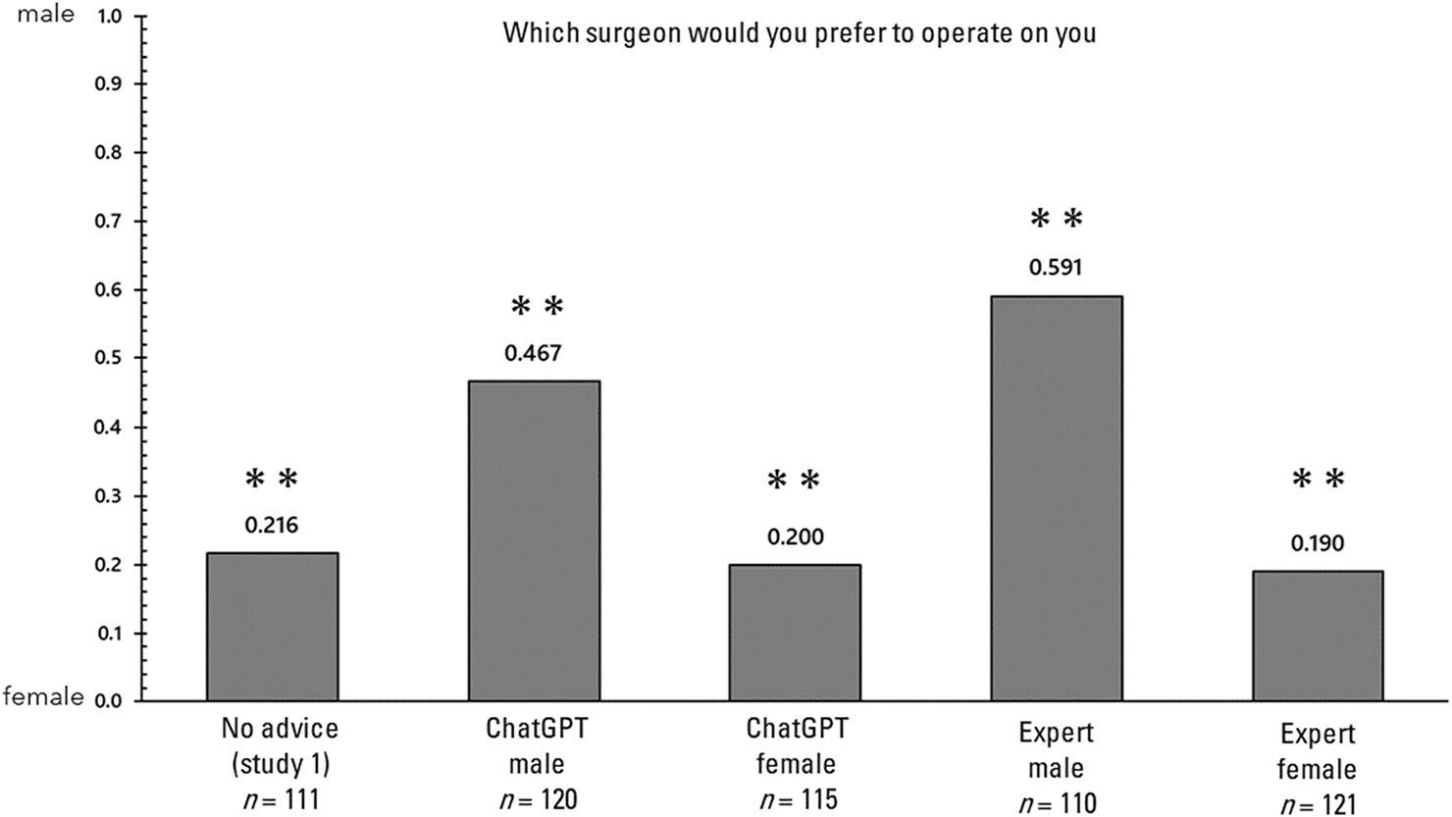
Response ratios for the gender stereotype task in Study 2. *p < 0.05, **p < 0.01.

First, the influence of advice on each of the four decision-making tasks is examined. In the analysis that follows, responses to each of the decision-making tasks will be examined, comparing the responses without advice in Study 1 with those in Study 2. It is important to note that in Study 1, unlike in Study 2, the relevant questionnaire was administered concurrently with several other questionnaires. Figure 2 displays the proportions of responses in the trolley problem (switch). A 2 × 5 chi-square test was conducted on these proportions to determine if there was any bias in the selection. In the chi-square test, the observed (measured) values in each condition are compared with the expected values to determine if there are significant differences or biases favoring the expected outcomes. This test helps to establish whether the distribution of observed values deviates significantly from what would be anticipated under the hypothesis of no effect or no difference. The results revealed a significant bias (χ^2^(4) = 39.198, *p* < 0.001, Cramer’s V = 0.264). Consequently, a residual analysis demonstrated that responses consistent with the advice were significantly more frequent only in the condition where advice was provided (*p*s > 0.05). Next, the proportions of responses in the trolley problem (bridge) are illustrated in Fig. 3. A 2 × 5 chi-square test was conducted on these proportions to ascertain whether they were biased, and no significant bias was detected (χ^2^(4) = 4.825, *p* = 0.306, Cramer's V = 0.092). Next, the proportions of responses in the delayed value discounting task are illustrated in Fig. 4. A 2 × 5 chi-square test was conducted on these proportions to determine whether there was bias, and significant bias was found (χ^2^(4) = 21.013, *p* < 0.001, Cramer’s V = 0.195). Consequently, a residual analysis was conducted, revealing significant bias in all three conditions (*p*s > 0.05), except for the no advice condition and the advising condition in which the AI recommended immediate rewards. Finally, the proportions of responses in the gender stereotyping task are presented in Fig. 5. A 2 × 5 chi-square test was performed on these proportions to determine whether they were biased, and a significant bias was found (χ^2^(4) = 69.896, *p* < 0.001, Cramer's V = 0.348). Therefore, a residual analysis was conducted, which indicated a predominant bias in all conditions (*p*s < 0.01).

Next, personal characteristics related to the susceptibility to AI and expert advice are examined. For all participants, the mean score for Personal Fear of Invalidity was 4.12 (SD = 0.91), the mean score for Trust in the social benefit of AI was 4.39 (SD = 0.85), and the mean score for Distrust in the fidelity of AI was 4.14 (SD = 0.87), with all scores ranging from 1 to 7. In Study 2, a score of 1 was assigned when the "responses obtained after the advice was presented" differed from the "responses obtained without the advice," and a score of 0 when they were the same. Subsequently, a logistic multiple regression analysis was conducted using the change in responses (change = 1, no change = 0) as the dependent variable for the group presented with AI advice (*n* = 906) and the group presented with expert advice (*n* = 908), respectively. The independent variables were age, gender (male = 1, female = 0), Personal Fear of Invalidity, Trust in the social benefit of AI, and Distrust in the fidelity of AI. Initially, only the effect of Personal Fear of Invalidity was significant when conducted for the group presented with AI advice (Table 1). A similar logistic multiple regression analysis was conducted for the group presented with expert advice, and similarly, only the effect of Personal Fear of Invalidity was significant (Table 2).

**Table 1:** Examination of factors affecting opinion change due to advice from AI.

Variables	Estimate	SE	Z	Wald statistic	p
Age	− 0.018	0.011	− 1.639	2.686	0.101
Sex	− 0.025	0.215	− 0.116	0.014	0.907
Personal fear of invalidity	0.303	0.124	2.438	5.943	0.015*
Trust in the social benefit of AI	0.116	0.129	0.897	0.804	0.370
Distrust in the fidelity of AI	0.122	0.124	0.987	0.975	0.324

McFadden R^2^ = 0.030, χ^2^ = 14.145, *p* = 0.015. *p < 0.05.

**Table 2:** Examination of factors influencing changes in opinion due to expert advice.

Variables	Estimate	SE	Z	Wald statistic	p
Age	− 0.000	0.011	− 0.020	0.000	0.984
Sex	0.094	0.213	0.442	0.196	0.658
Personal fear of invalidity	0.241	0.119	2.016	4.066	0.044*
Trust in the social benefit of AI	− 0.036	0.121	− 0.295	0.087	0.768
Distrust in the fidelity of AI	− 0.034	0.122	− 0.275	0.076	0.783

McFadden R^2^ = 0.007, χ^2^ = 4.570, *p* = 0.471. *p < 0.05c.

### Discussion

In Study 2, the impact of advice presented as ChatGPT or from an expert on decision-making was examined. The findings indicated that in the trolley problem (switch), no bias was observed in the absence of advice, but when advice was provided by an AI or an expert, responses were biased in the direction consistent with the advice. Conversely, in the trolley problem (bridge), the advice did not alter the response bias. This trend diverges from that of Krügel et al.^13^, upon which this study was based, and is a result that was unveiled only because a controlled condition was established. The reason why advice did not affect decision-making in the trolley problem (bridge) is unclear, but it may be that logical advice does not impact decision-making driven by negative emotions^24^. In the delayed value discounting task, only the advice from the AI endorsing immediate rewards did not affect decision-making, while the advice from the AI endorsing delayed rewards and the advice from experts both altered decision-making in a direction consistent with their content. Further study is required to determine why the advice from the AI endorsing immediate rewards did not influence decision-making, but participants may have distrusted the AI's recommendation of the lower-priced option. Finally, in the gender stereotyping task, the results showed that in the absence of advice, the selection rate of female surgeons was higher, but when a male surgeon was recommended, the selection rate of male surgeons increased, regardless of whether the advisor was ChatGPT or a expert. This suggests that stereotypes may be reinforced not only by experts but also by ChatGPT discourse. On the other hand, advice in the direction of stereotype mitigation, whether given by ChatGPT or by experts, did little to change the ratio of no advice. This may indicate that logical advice was still challenging to influence decision-making made with negative feelings^27^ caused by stereotypes.

## General discussion

The present study builds upon the finding by Krügel et al.^13^ that human decision-making can be influenced by advice from ChatGPT, extending it from the following three perspectives: (1) the control condition was found to vary in two directions, (2) decision-making other than moral judgments was also influenced by advice from ChatGPT, and (3) individual characteristics related to susceptibility to advice from ChatGPT were identified.

First, a study of Japanese subjects, relying on the methodology of Krügel et al.^13^, demonstrated that decision-making in moral judgments was influenced by advice from ChatGPT and experts. However, this was only the case in the switch condition, where responses were split roughly in half in the absence of advice, and there was no effect of advice in the bridge condition, where many responded negatively in the absence of advice (i.e. five people were left to die). This is a finding that became clear only because this study set up a control condition. Although further study is needed to determine why the advice was not effective in the bridge condition, Japanese may have been particularly resistant to being verbally persuaded to respond to the negative feelings^24^ that arise from pushing another person off a bridge. In fact, it is possible that emotional factors are more important in persuasion in Asia, including Japan, than in the West^28^. In the future, it will be necessary to examine cultural differences in persuasion by ChatGPT, as well as the content that is likely to be effective in each culture.

Second, this study also conducted a delayed value discounting task and a gender stereotyping task to determine whether advice from ChatGPT influenced decision-making outside of moral judgments. The results showed that in the delayed value discounting task, expert advice influenced participants' responses in both content areas, while advice from ChatGPT had no effect on the recommendation of low immediate rewards. It is unclear why the AI's recommendation of immediate rewards had no effect, and the reasons for this are still pending further investigation. In the gender stereotyping task, the advice influenced decision-making in the direction that promoted stereotyping (i.e. recommending a male surgeon with fewer years of experience), but not in the direction that mitigated stereotyping (i.e. recommending a female surgeon with more years of experience). This was true for both ChatGPT advice and expert advice, suggesting that stereotypes can be reinforced even in ChatGPT discourse. Because stereotype mitigation is not easy^29^, the advice may not have had an effect.

Third, this study also examined individual characteristics related to susceptibility to influence from advice. The results showed that age and gender had no effect, only Personal Fear of Invalidity, and Trust for AI had no effect. In other words, the results indicated that individuals who lack confidence in their own decision-making are more likely to be influenced by advice from AI and experts, and that their reliance on advice from AI is not due to trust in AI. It is possible that people do not perceive a significant difference between advice from experts and advice from ChatGPT, and they consider both as reference points when they are uncertain.

This study extends the findings by Krügel et al.^13^ and is particularly significant given the increasing integration of AI, such as ChatGPT, into our lives. However, this study has the following limitations. First, this study was conducted solely on Japanese subjects. The results of this study also suggest that there may be cultural differences in susceptibility to advice, so future research will need to include multicultural comparisons. Second, this study found differences in the type of advice given and the influence of its content depending on the type of decision-making. The present study extended the findings of Krügel et al.^13^ by exploring decision-making in domains beyond morality. However, further examination of decision-making in additional domains is warranted. In the future, it will be crucial to delineate more specifically which types of decision-making are influenced by advice from ChatGPT, as well as those that are not, and to understand the reasons behind these effects. Additionally, it will be important to consider variations in the impact of such advice across different genders and age groups of participants.

### Consent to participate

Informed consent was obtained from all subjects for participation.

## Data Availability

The data that support the findings of this study and materials are openly available in Open Science Framework at https://osf.io/9zu83/?view_only=dd551f6cce034f8c8af3d29d4ba68a52.
